# DANCR promotes HCC progression and regulates EMT by sponging miR‐27a‐3p via ROCK1/LIMK1/COFILIN1 pathway

**DOI:** 10.1111/cpr.12628

**Published:** 2019-04-30

**Authors:** Dan Guo, Yarui Li, Yifei Chen, Dan Zhang, Xin Wang, Guifang Lu, Mudan Ren, Xinlan Lu, Shuixiang He

**Affiliations:** ^1^ Department of Gastroenterology The First Affiliated Hospital of Xi'an Jiaotong University Xi'an Shaanxi China

**Keywords:** differentiation antagonizing nonprotein coding RNA, epithelial‐mesenchymal transition, hepatocellular carcinoma development, LIM domain kinase 1, miR‐27a‐3p

## Abstract

**Objectives:**

This research aims to verify that the long non‐coding RNA differentiation antagonizing nonprotein coding RNA (LncRNA DANCR) could modulate the proliferation and metastasis of hepatocellular carcinoma (HCC), and it thus may work as a novel biomarker to render new orientation for early diagnosis and clinical therapy of HCC.

**Materials and methods:**

Firstly, qRT‐PCR was used to detect the expression of genes including LncRNA DANCR and miR‐27a‐3p. Next, MTT assay, Ethynyldeoxyuridine (EdU) analysis and clone formation assay were used for investigating cell growth and proliferation. Meanwhile, transwell assay and wound healing assay were applied to evaluate the capacity of cell metastasis and motility, respectively. In addition, bioinformatic analysis and dual‐luciferase reporter assay were applied to analyse molecular interaction. Next, we conducted immunofluorescence and Western blot for mechanic investigation. Last but not the least, xenograft tumours in nude mice were built by subcutaneously injecting Hep3B cells stably transfected with sh‐NC and sh‐DANCR to detect proliferation and SMMC‐7721 cells stably transfected with sh‐NC and sh‐DANCR to investigate metastasis.

**Results:**

The results of qRT‐PCR and bioinformatic analysis revealed the high expression of DANCR in HCC. DANCR accelerated proliferation and metastasis of HCC cells and the knockdown of DANCR had the opposite effect. Meanwhile, xenograft tumours in sh‐DANCR group grow slower and have smaller volumes compared with negative control group. Next, the antineoplastic effect of miR‐27a‐3p on cell growth and motility of HCC was confirmed. In addition, we clarified that DANCR acted as a ceRNA to decoy miR‐27a‐3p via mediating ROCK1/LIMK1/COFILIN1 pathway. In the end, we validated that DANCR/miR‐27a‐3p axis regulates EMT progression by cell immunofluorescence and Western blot.

**Conclusions:**

In a word, DANCR promotes HCC development and induces EMT by decoying miR‐27a‐3p to regulate ROCK1/LIMK1/COFILIN1 pathway.

## 1 INTRODUCTION

Cancer‐related mortality caused by hepatocellular carcinoma (HCC) has been increasing in the past few years with more than 700 000 deaths per year,^1^, ^2^ despite that the diagnosis and treatment of HCC have been greatly advanced. Therefore, it's urgent to explore potential biomarkers and in‐depth oncological mechanism of HCC progression, which contribute to early diagnosis and effective therapy.

With the development of RNA sequencing technologies and bioinformatics, Genome Projects have revealed that >90% of the human genome belong to non‐coding RNAs (ncRNAs), which now are highlighted in scientific field of physical and pathological progress including differentiation, metabolism, proliferation, metastasis,^3^ in spite of not being translated into proteins.^3^, ^4^, ^5^ Cut‐off by the length of 200 bp, ncRNAs are divided into small non‐coding RNAs (sncRNAs) and long non‐coding RNAs (lncRNAs), either of which is closely relative to biology and oncology.^3^ LncRNAs interfere gene expression in transcriptional or post‐transcriptional process by direct or indirect ways. For instance, lncRNA HOXD‐AS1 promotes osteosarcoma by recruiting the enhancer of zeste homolog 2 (EZH2), which binds to the promoter of P57 to inhibit p57 expression.^6^ LncRNA HCP5 functions as a ceRNA of miR‐22‐3p, miR‐186‐5p and miR‐216a‐5p, which activate ST6GAL2 and promote the development of follicular thyroid carcinoma.^7^

Long non‐coding RNA differentiation antagonizing nonprotein coding RNA (DANCR), which was first described as a lncRNA blocking differentiation of the epidermal progenitor cells, has been studied in various tumour progression in recent years.^8^, ^9^ As reported, lncRNA DANCR promoted osteosarcoma proliferation, migration and invasion, as well as mediated cancer stem cells features by modulating AXL expression via miR‐33a‐5p sponge.^10^ As for hepatocellular carcinoma, DANCR stimulated stemness features by regulating CTNNB1^11^ and promoted liver cancer cells proliferation and metastasis as an oncogene.^12^ However, to provide us a new research orientation and gain insights into its role in HCC progression, more detailed mechanism of the role of DANCR in HCC are still needed.

To indicate the overexpression of DANCR in HCC tissues, the results analysed from various database were combined and the function of DANCR on HCC development in vivo and vitro was further detected. We investigated the expression level of DANCR in HCC cell lines (MHCC‐97H, MHCC‐97L, HCC‐LM3, Hep‐G2, Hep‐3B, Huh7 and SMMC‐7721) compared with the immortalized, normal human hepatic cell LO2. Our study indicated that DANCR was overexpressed and positively correlated with the expression of LIM domain kinase 1 (LIMK1). Meanwhile, we surprisingly found that miR‐27a‐3p could compete with DANCR for binding the same site on the transcript of LIMK1. Therefore, we propose that the DANCR/miR‐27a‐3p/LIMK1 axis may have great influence in hepatocellular carcinoma progress.

## 2 MATERIALS AND METHODS

### 2.1 Bioinformatic analysis

starbase 3.0^13^, ^14^ and gepia 15 were used for analyzing the expression of genes including DANCR, miR‐27a‐3p and gene co‐expression. Kaplan‐Meier and log‐rank analyses were used for survival analysis. The downstream genes and detailed binding site of miR‐27a‐3p were predicted by targetscan 7.1.^16^ annolnc 17 was applied to comprehensively understand DANCR and acknowledge the binding site between DANCR and miR‐27a‐3p.

### 2.2 Cell culture

All human HCC cells involved and immortalized human hepatic cell LO2 were purchased from the Type Culture Collection of the Chinese Academy of Sciences. All cells were cultured in DMEM/high glucose (Hyclone), supplemented with 10% foetal bovine serum (FBS, Gibco) and 100 μg/mL streptomycin and 100 U/mL penicillin (Hyclone), and maintained at 37°C in humidified incubator with 5% CO_2_.

### 2.3 Plasmid construction and cell transfection assay

The sequences of small interfering RNA against DANCR (Genepharma) and miRNA mimics or inhibitor for miR‐27a‐3p have been all listed in Table 1. For stable transfection, shRNA against DANCR, which inserted siRNA into the GV112 vector, was designed by Genechem. Meanwhile, puromycin was used for 2 weeks to select stably transfected cell lines. DANCR was overexpressed by transfecting pCMV vector with DANCR sequence cloning into it. Transfection assay was conducted according to the instruction of Lipofectamine 2000 (Invitrogen) when the cells reached approximately 60%‐80% confluence.

**Table 1:** Sequences of siRNA and miRNA mimics or inhibitor used in this study

Gene		Sequence
Negative control	Sense	5′‐UUCUCCGAACGUGUCACGUTT‐3′
	Antisense	5′‐ACGUGACACGUUCGGAGAATT‐3′
siDANCR		GCUGGUAUUUCAAUUGACUTT'
		AGUCAAUUGAAAUACCAGCTT
Negative control	Sense	5′‐UUCUCCGAACGUGUCACGUTT‐3′
	Antisense	5′‐ACGUGACACGUUCGGAGAATT‐3′
Human miR‐27a‐3p mimics		UUCACAGUGGCUAAGUUCCGC
		GGAACUUAGCCACUGUGAAUU
MiRNA inhibitor N.C		5′‐CAGUACUUUUGUGUAGUACAA‐3′
Human miR‐27a‐3p inhibitor		GCGGAACUUAGCCACUGUGAA

### 2.4 The isolation of RNA and quantitative real‐time PCR (qRT‐PCR)

All RNAs were extracted according to the instruction of Trizol Reagent (Invitrogen). Then the cDNAs were synthesized following the protocol of PrimeScript™ RT Master Mix (Takara). Quantitative real‐time PCR was conducted using SYBR Premix Ex Taq™ II (Takara) on Thermal Cycler CFX6 System (BioRad). The relative expression of genes was calculated by the 2^−ΔΔCt^ formula. U6 and β‐actin were used as internal reference. The primers involved were presented in Table 2.

**Table 2:** Primers of genes in this research for qRT‐PCR

Gene	Sequences of primer
DANCR	Forward: GCCCTTGCCCAGAGTCTTCC
	Reverse: CTATTTCTGAATATACAGCCAAGACAAGTGGC
LIMK1	Forward: GCTTCTACCTCGTGGCTTGTG
	Reverse: GCTGCTCTGCTCACTGTCAC
β‐actin	Forward: ATCGTGCGTGACATTAAGGAGAAG
	Reverse: AGGAAGGAAGGCTGGAAGAGTG
hsa‐miR‐27a‐3p	
RT primer: GTCGTATCCAGTGCAGGGTCCGAGGTATTCGCACTGGATACGACGCGGAA	
Forward: GCGCGTTCACAGTGGCTAAG	
Reverse: AGTGCAGGGTCCGAGGTATT	
U6	
RT primer: GTCGTATCCAGTGCAGGGTCCGAGGTATTCGCACTGGATACGACAAAATA	
Forward: AGAGAAGATTAGCATGGCCCCTG	
Reverse: AGTGCAGGGTCCGAGGTATT	

### 2.5 Proliferation assays

#### 2.5.1 MTT assay

Cell viability was assessed by MTT (3‐(4, 5‐dimethylthiazol‐2‐yl)‐2, 5‐diphenyltetrazolium bromide) assay. Firstly, cells were seeded into 96‐well plates at a density of 6000 cells per well. 24 hours after seeding, transfection was conducted as the reagent's protocol. After additional 24, 48, 72, 96 hours, 10 μL of 5 mg/mL MTT was added into each well and then cultured for 4 hours in incubator. The supernatant was then discarded and 150 μL of DMSO was added to dissolve the crystal. The optical density (OD) was measured by EnSpire Multimode Plate Reader (PerkinElmer) at 490 nm.

#### 2.5.2 Ethynyldeoxyuridine (EdU) analysis

Cells were seeded into 96‐well plate and transfected. 48 hours after transfection, EdU staining was proceeded as the instruction of EdU kit (KeyGEN BioTECH). The EdU‐positive cells were observed and counted under the fluorescence microscopy.

#### 2.5.3 Clone formation assay

After 24 hours of transfection, cells were planted into 6‐well plate (500 cells per well). After about 2 weeks, when clones formed by single cell possessed at least 50 cells, the clones were fixed with methanol and then stained with 0.1% crystal violet. The clones were counted for statistical analysis after airing.

### 2.6 Metastasis assay

#### 2.6.1 Transwell assay

After 48 hours of transfection, cells were, respectively, seeded into of 8 μm pore size transwell 24‐well chambers (Merck Millipore) coated with Matrigel (BD Biosciences) for invasion assay and non‐coated chambers for migration. On the top of chamber is serum‐free DMEM medium and below containing 10% FBS. After incubation for at least 24 hours, wiped off the non‐invaded or non‐migrated cells gently on the top of chambers. Then the chambers were fixed with 95% ethyl alcohol for 10 minutes, followed by crystal violet staining for 15 minutes and washed in PBS. Stained cells were pictured and counted under the inverted microscope.

#### 2.6.2 Wound healing assay

Appropriate cell density is required which need to attain 90% after 24 hours of transfection in 6‐well plates. Wound was scratched by 10 μL sterile tip and then washed off the floated cells. Under the microscope took photographs for the wound, respectively, at 0, 24 and 48 hours.

### 2.7 Western blot analysis

All proteins were isolated after transfection for 72 hours as the protocol of radioimmunoprecipitation assay (RIPA) lysis buffer (Pierce) which was added proteinase and phosphatase inhibitors in advance. Protein samples were loaded for electrophoresis (5% gel for concentration and 10% for separation) and then transferred onto 0.45 μm or 0.22 μm pore size PVDF membrane (Merck Millipore). After blocking with 5% defatted milk gently for 1 hour, the membrane was incubated with corresponding primary antibodies (Table 3) at 4°C overnight. The next day washed the membrane and then incubated with secondary antibodies (Zhuangzhi Biology, dilution rate of 1:5000) for 1 hour at room temperature. Proteins bands were detected by using ECL immunoblotting kit (Millipore). For convenience, the raw data of Western blot were supplied in Figures S1‐S3 for bands involved in Figures 5J, 7F and 8 in sequence.

**Table 3:** details of primary antibodies applied in this study

Gene specificity	Manufacture of primary antibody	Dilution rate		Specificity
		WB	IF	
ROCK1	Cell Signaling Technology	1:1000	—	Rabbit
LIMK1	Abcam	1:1000	—	Rabbit
Cofilin1	Cell Signaling Technology	1:1000	—	Rabbit
p‐cofilin1	Cell Signaling Technology	1:800	—	Rabbit
E‐cadherin	Cell Signaling Technology	1:1000	1:200	Rabbit
N‐cadherin	Cell Signaling Technology	1:1000	1:200	Rabbit
Vimentin	Cell Signaling Technology	1:1000	1:100	Rabbit
GAPDH	CWBIO	1:5000	—	Mouse

### 2.8 Dual‐luciferase reporter assay

Firstly, we constructed the plasmids which were used for co‐transfection of dual‐luciferase reporter assay. Plasmid GV208‐DANCR‐wt (Genechem) was constructed by inserting the sequence of DANCR into the GV208 vector, and plasmid GV208‐DANCR‐mut (Genechem) was established by inserting DANCR sequence with the miR‐27a‐3p binding site muted by site‐specific mutagenesis. The day before transfection, cells were seeded into 96‐well plate at approximately 60%‐80% confluence. 200 ng plasmid GV208‐DANCR‐wt or GV208‐DANCR‐mut, together with 0.25 μL of 5pmol miR‐27a‐3p NC, mimics, inhibitor NC or inhibitor, were co‐transfected with Lipofectamine 2000 (Invitrogen) according to the manufacture's protocol. The luciferase activity was detected using the Dual Luciferase Assay Kit (Promega). The Renilla luciferase activity was regarded as control.

### 2.9 Cell immunofluorescence

Cells were seeded on the coverslips for 24 hours to 50%‐60% confluence. Then the cells were fixed by 4% paraformaldehyde for 15 minutes, and 0.5% Triton‐100 was used for permeability for 20 minutes at room temperature. Next, the coverslips were blocked with 10% goat serum at room temperature for 30 minutes. Following the corresponding primary antibodies of appropriate dilution by 10% goat serum (Table 3) were incubated on the coverslips for overnight at 4°C. The next day after washed for three times using PBS, the cells were incubated with secondary antibody (Zhuangzhi Biology, dilution rate 1:100) for 1 hour. After washing, DAPI was applied for nucleus staining. Images were collected using the fluorescent inverted microscope by putting the coverslips reverse.

### 2.10 Xenograft tumour models in nude mice

Male athymic BALB/C nude mice (4 weeks old) were purchased from the Central Laboratory of Animal Science, Xi'an Jiaotong University, China. 200 μL of Hep3B cell suspension with approximately 5 × 10^6^ cells, which were stably transfected with sh‐NC or shDNACR, was subcutaneously injected for detecting tumour growth. Tumour size was measured every 4 days. One month after injection, all experimental mice were sacrificed. Meanwhile, 100 μL of SMMC‐7721 suspension with about 3 × 10^6^ cells, which were stably transfected with sh‐NC or shDNACR, was intravenously injected for pulmonary metastasis. Those mice were sacrificed 2 months after injection. Specimens were collected and stored at 4% formaldehyde for further detection. The animal experiment was thoroughly operated according to the Guide for the Care and Use of Laboratory Animals of the National Institutes of Health and was authorized by the medical ethics committee of the First Affiliated Hospital of Xi'an Jiaotong University.

### 2.11 Statistical analysis

All data were analysed using spss 23.0 (IBM, SPSS) and graphpad prism 7.0 (GraphPad Software, Inc). The difference between two groups was compared by Student's *t* test and Pearson correlation analysis was applied for correlation of genes involved. A value of two‐tailed *P* < 0.05 is considered as statistically significant while *P* < 0.01 is very significant.

## 3 RESULTS

### 3.1 DANCR is overexpressed in HCC and predicts poor prognosis in HCC patients

**Figure 1:**
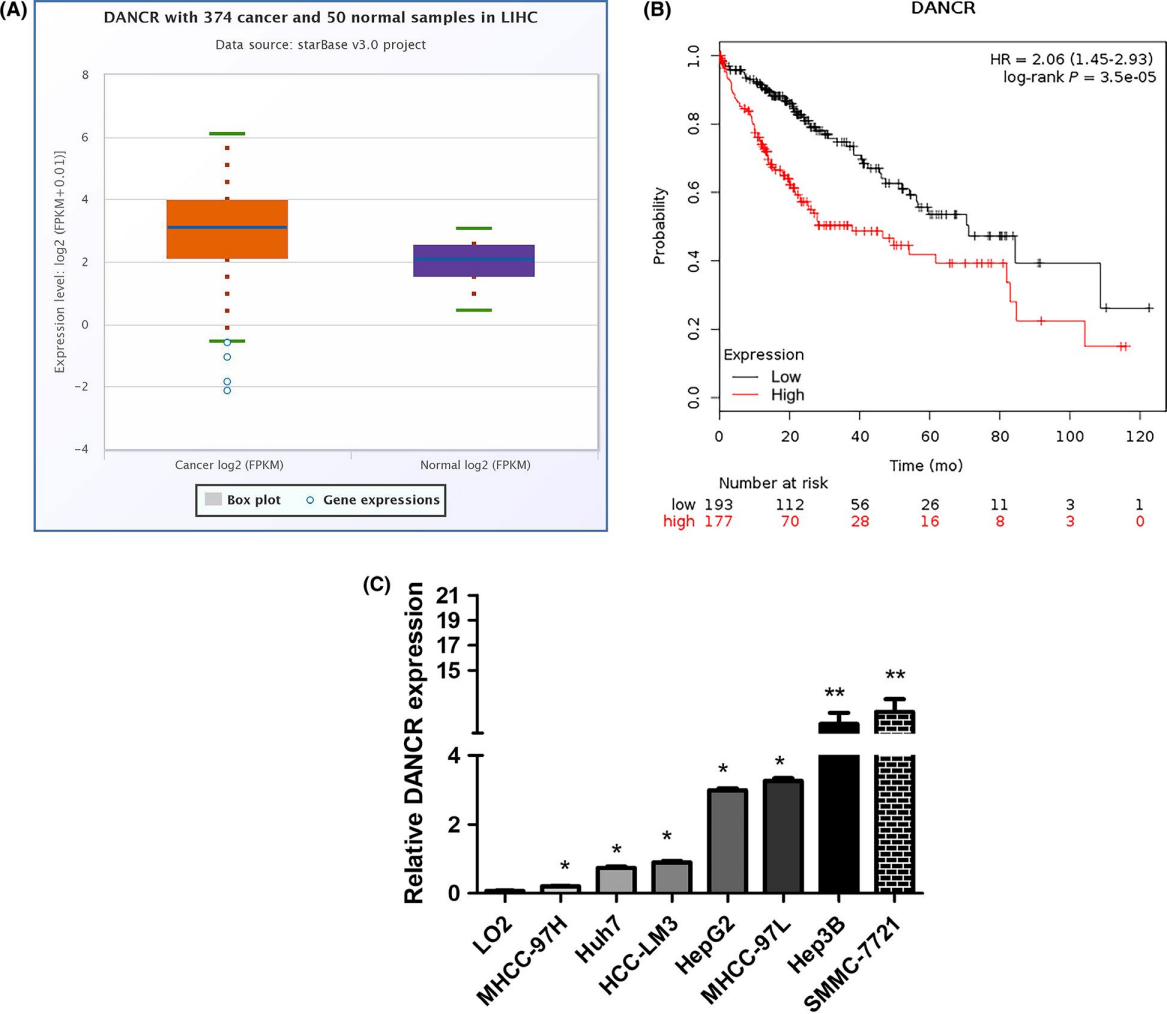
DANCR is overexpressed in HCC and predicts poor prognosis in HCC patients. A, The analysis of starbase 3.0 in HCC samples showed the high expression of DANCR in HCC *P* < 0.05. B, Kaplan‐Meier plotter analysis for overall survival of HCC patients which were divided into high expression of DANCR and low. The results indicated that patients of low expression of DANCR had better prognosis than those of high expression. *P* < 0.01. C, qRT‐PCR was applied to analyse the expression of DANCR in various HCC cells. Compared with LO2, DANCR was also overexpressed in HCC cells. *P < 0.05, **P < 0.01. All data were collected by repeating three‐time experiments which were mutually independent as mean ± SD

Bioinformatic analysis indicated that DANCR was overexpressed in HCC (Figure 1A, starbase 3.0, *P < *0.05). Kaplan‐Meier plotter analysis suggested that the overall survival of patients with high expression of DANCR was shorter than those with low expression (Figure 1B, *P* < 0.01). To further verify the results of bioinformatic analysis, we detected the expression of DANCR in HCC cells including MHCC‐97H, Huh7, HCC‐LM3, HepG2, MHCC‐97L, Hep3B, SMMC‐7721 and immortalized human hepatic cell LO2 by qRT‐PCR. In accordance with the results above, the high expression of DANCR was also investigated in HCC cells (Figure 1C). In all, we speculate that DANCR may act an important role in HCC progression.

### 3.2 Suppression of DANCR inhibits growth and metastasis of HCC cells

**Figure 2:**
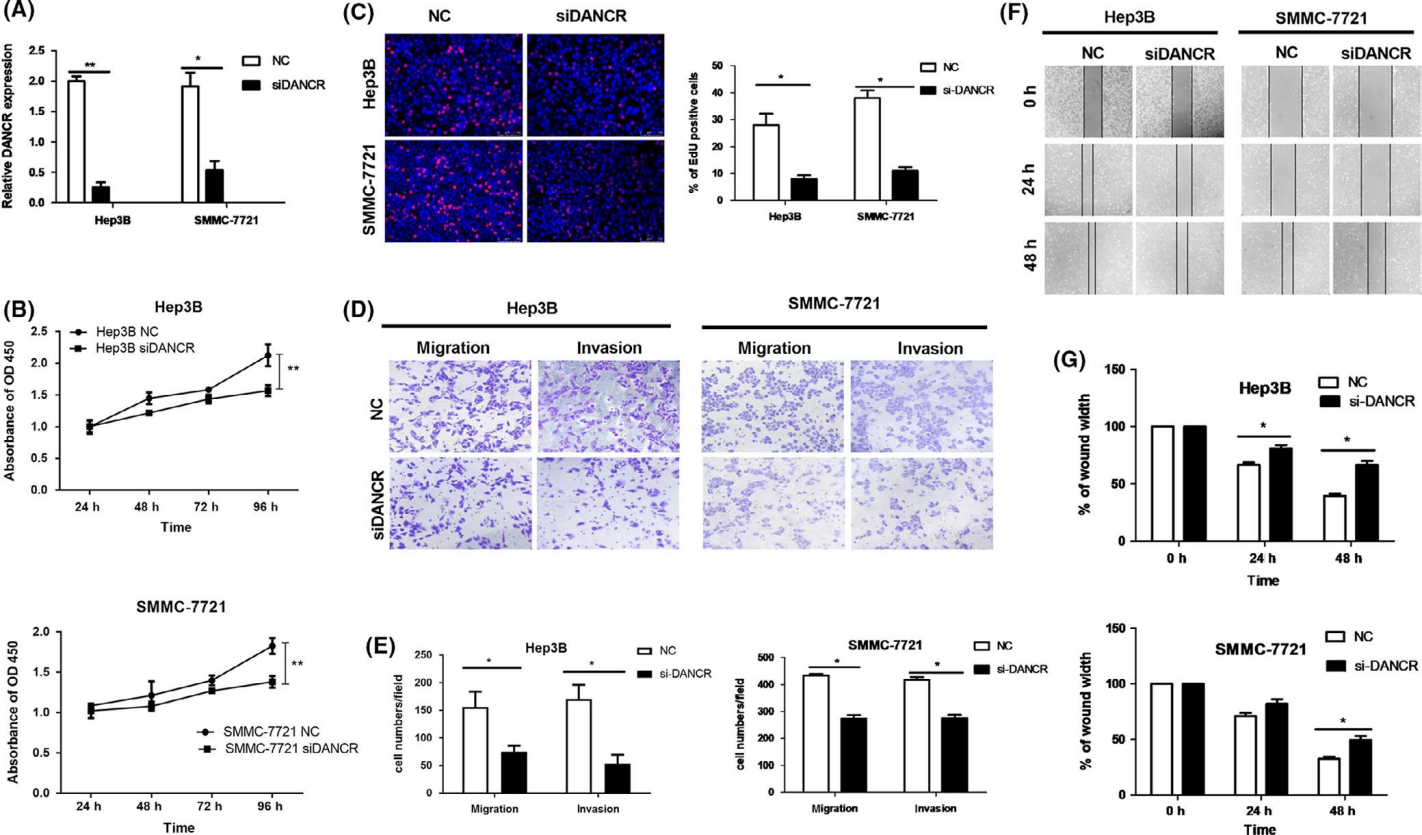
Suppression of DANCR inhibits growth and metastasis of HCC cells. A, qRT‐PCR was used to analyse the efficiency of knockdown of DANCR in Hep3B and SMMC‐7721 by a specific siRNA against DANCR, and the result showed that the expression of DANCR was obviously attenuated. *P < 0.05, **P < 0.01. B, When DANCR was knockdown, HCC cells grew slower than NC group in MTT assay **P < 0.01. C, In EdU assay, the percentage of EdU‐positive cells in siDANCR group clearly declined compared with NC group, which further verified the conclusion of the MTT assay. *P < 0.05. D and E, Just as the result of transwell assay shown, When DANCR was knocked down, the migration and invasion of HCC cells were repressed. *P < 0.05. F and G, In wound healing assay, the motility of HCC cells was reduced in siDANCR group when compared with NC group. *P < 0.05. All data were collected by repeating three‐time experiments which were mutually independent as mean ± SD

In order to comprehensively acknowledge the function of DANCR in HCC progression, a specific siRNA against DANCR gene transcript was designed to knockdown DANCR in Hep3B and SMMC‐7721, which had higher endogenous DANCR expression, and qRT‐PCR verified that the expression of DANCR was obviously decreased by this siRNA (Figure 2A). Next, we further conducted functional experiment. The MTT assay displayed that downregulation of DANCR significantly repressed cell growth over time compared with negative control (NC) group (Figure 2B). Similar to the result of MTT assay, the percentage of EdU‐positive cells in siDANCR group was less than the NC group (Figure 2C). Transwell assay indicated that the cell capacity of migration and invasion was reduced when DANCR was knocked down (Figure 2D,E). Consistent with the results above, knockdown of DANCR obviously eliminated the motility of HCC cells compared with NC group in wound healing assay (Figure 2F,G). In a word, downregulation of DANCR represses HCC cells proliferation and metastasis.

### 3.3 Overexpression of DANCR promotes growth and metastasis of HCC cells

**Figure 3:**
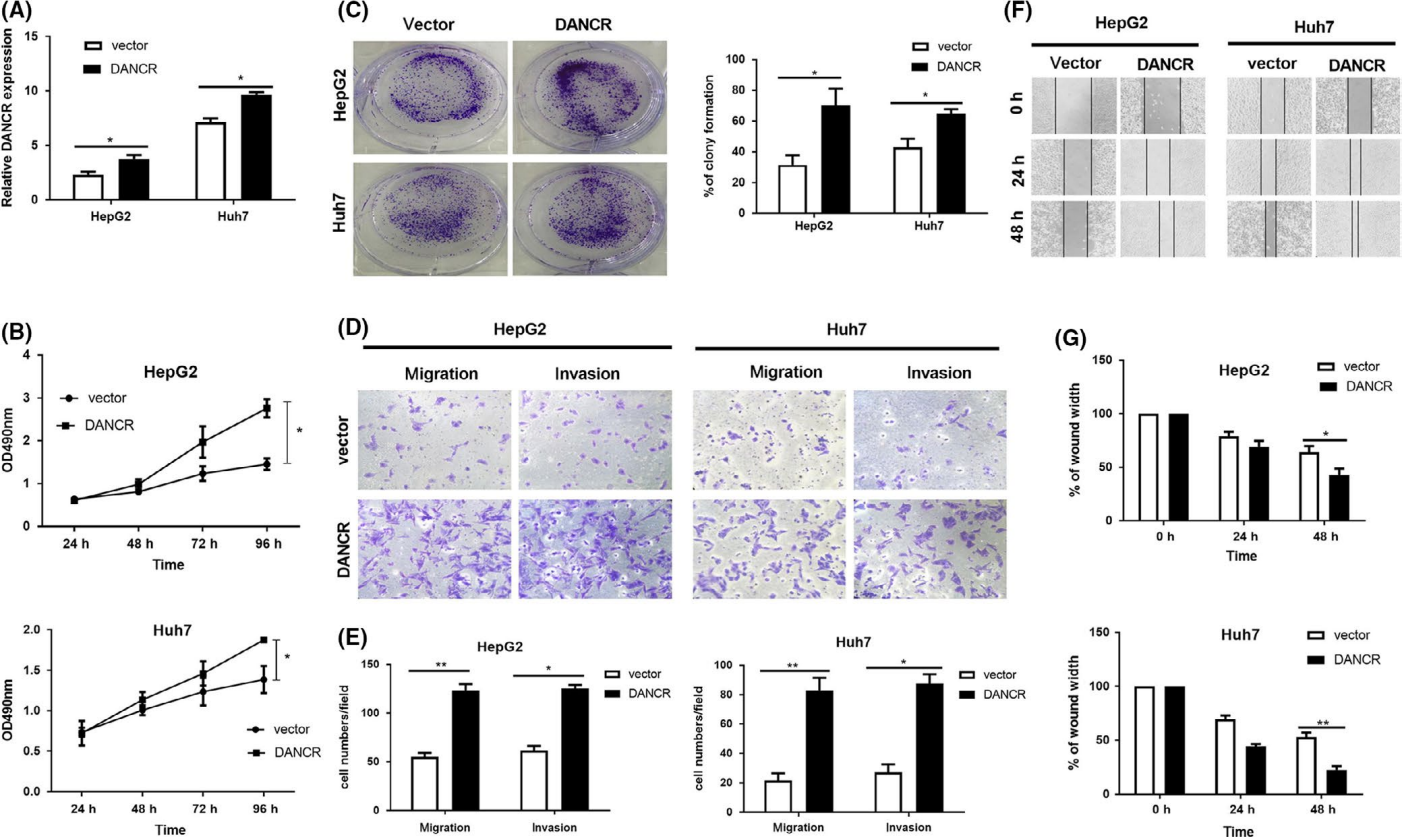
Overexpression of DANCR promotes growth and metastasis of HCC cells. A, The efficiency of DANCR overexpression by transfecting with pCMV‐DANCR vector was analysed by qRT‐PCR and proved to be effective. *P < 0.05. B, DANCR overexpression promoted HCC cells proliferation over time in MTT assay. *P < 0.05. C, Clone formation was accelerated in DANCR group compared with empty vector group. *P < 0.05. D and E, In transwell assay, the cell number of migrated and invaded apparently increased when DANCR was overexpressed. *P < 0.05, **P < 0.01. F and G, Similarly, DANCR improved the motility of HCC cells compared with vector group in wound healing assay. *P < 0.05. All data were collected by repeating three‐time experiments which were mutually independent as mean ± SD

To further verify the function of DANCR in HCC, we transfected pCMV‐DANCR vector into HepG2 and Huh7 to overexpress DANCR, and the transfection effect was tested by qRT‐PCR (Figure 3A). In this part, we conducted MTT assay and clone formation assay to assess the influence on cell proliferation. The growth curve produced by MTT assay showed that DANCR upregulation significantly accelerated HCC cell growth (Figure 3B). Consistently, the clone formation number in DANCR group was increased compared with empty vector group (Figure 3C), which also indicated that DANCR promotes HCC cells proliferation. Meanwhile, transwell assay and wound healing assay were applied to detect the effect on metastasis. Overexpression of DANCR clearly augmented the ability of migration and invasion in transwell assay (Figure 3D,E). And in wound healing assay, DANCR expedites the mobility of HepG2 and Huh7 (Figure 3F,G). In general, DANCR may act as an oncogene in HCC development.

### 3.4 Knockdown of DANCR represses tumour growth and lung metastasis in vivo

**Figure 4:**
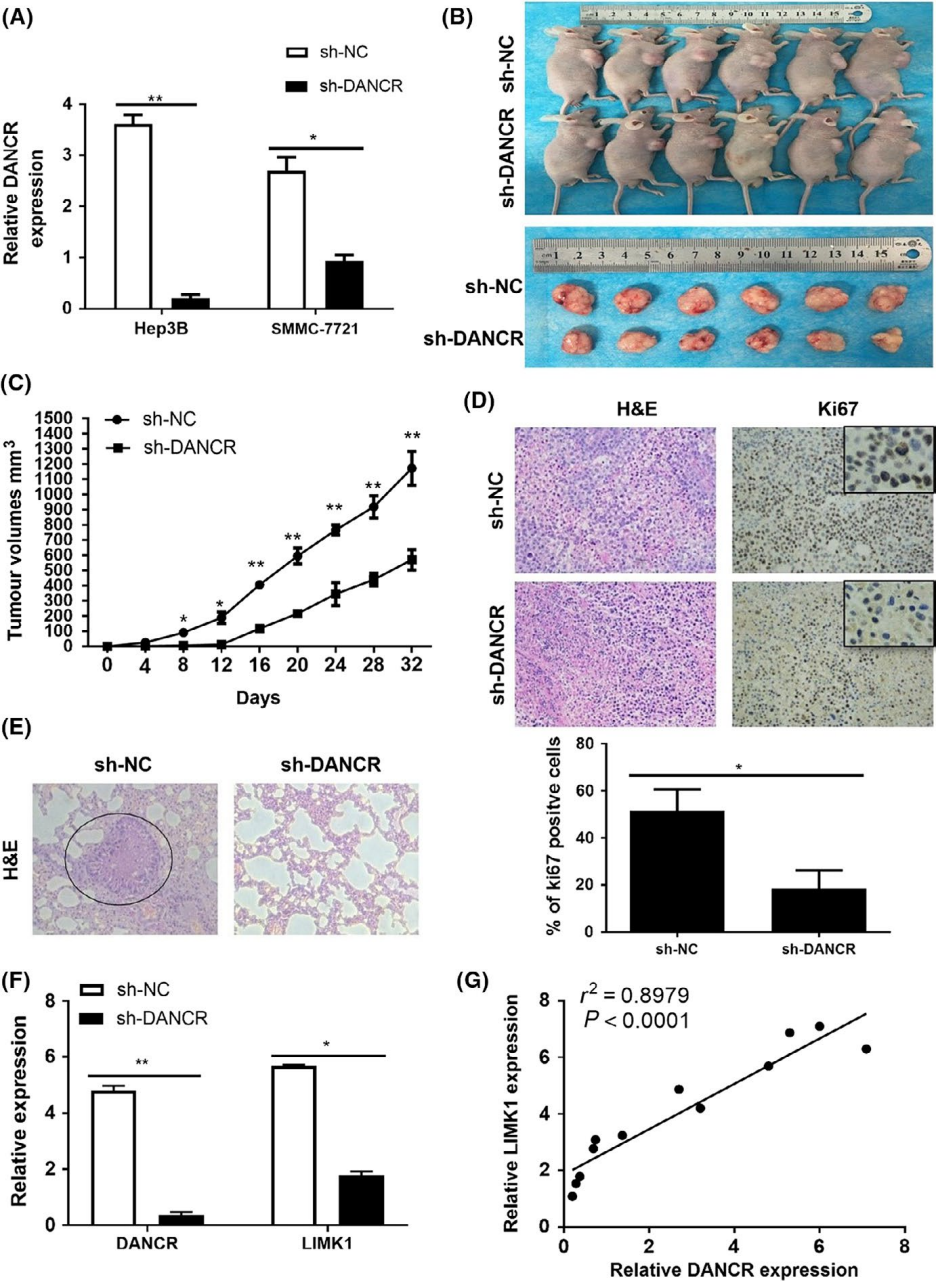
Knockdown of DANCR depresses tumour growth and lung metastasis in vivo. A, The transfection efficiency of HCC cells stably transfected with sh‐DANCR was detected by qRT‐PCR, and the result showed that the expression of DANCR is evidently reduced. *P < 0.05, **P < 0.01. B, The picture of nude mice which were sacrificed to form xenograft tumour models by subcutaneously injecting HCC cells Hep3B stably transfected with sh‐DANCR. C, The growth curve of xenograft tumour volumes was also applied to assess the function of DANCR in vivo. The volumes of sh‐DANCR xenograft tumours grew slower than sh‐NC group. *P < 0.05, **P < 0.01. D, Representative pictures of H&E and IHC staining of ki67 for tumour models. It was obvious that the expression of Ki67 was decreased when DANCR was knockdown. *P < 0.05. E, Classic pictures of H&E for lung metastasis models. There were much more lung metastasis nodules in sh‐NC group while rare in sh‐DANCR group. F, QRT‐PCR was used for detecting the expression of DANCR and LIMK1 in xenograft tumours and the result showed the lower expression of DANCR and LIMK1 in sh‐DANCR group in contrast to the NC group. *P < 0.05, **P < 0.01. G, Pearson correlation analysis indicated the expression of LIMK1 was positively correlated with DANCR in xenograft tumours. *r* ^2^ = 0.8979, *P* < 0.0001. All data were collected by repeating three‐time experiments which were mutually independent as mean ± SD

We further confirmed the oncogenic effect of DANCR in vivo by subcutaneously injecting human HCC cells Hep3B transfected with sh‐DANCR into the nude mice to form xenograft models, and HCC cells SMMC‐7721 stably transfected with sh‐DANCR were intravenously injected for 2 months to investigate lung metastasis. Firstly, the efficiency of stably transfected cells was confirmed by qRT‐PCR. As the result shown in Figure 4A, the expression of DANCR was knocked down when transfected with sh‐DANCR. Identically, xenograft tumour models in sh‐DANCR group grew slower compared with the sh‐NC group (Figure 4B,C). Besides, downregulation of DANCR distinctly reduced the expression of ki67 in contrast to sh‐NC group (Figure 4D). As for lung metastasis models, there were clearly a greater number of lung metastatic nodules in sh‐NC group in contrast to sh‐DANCR group. (Figure 4E). In addition, the expression of DANCR and LIMK1 was investigated in xenograft tumours by qRT‐PCR. As shown in Figure 4F, both DANCR and LIMK1 were knocked down in sh‐DANCR group compared with sh‐NC group. Then, Pearson correlation analysis indicated the positive correlation between DANCR and LIMK1 in xenograft tumour models (Figure 4G). In brief, DANCR knockdown distinctly suppresses HCC growth and lung metastasis in vivo models.

### 3.5 DANCR acts as a sponge of miR‐27a‐3p to regulate LIMK1

**Figure 5:**
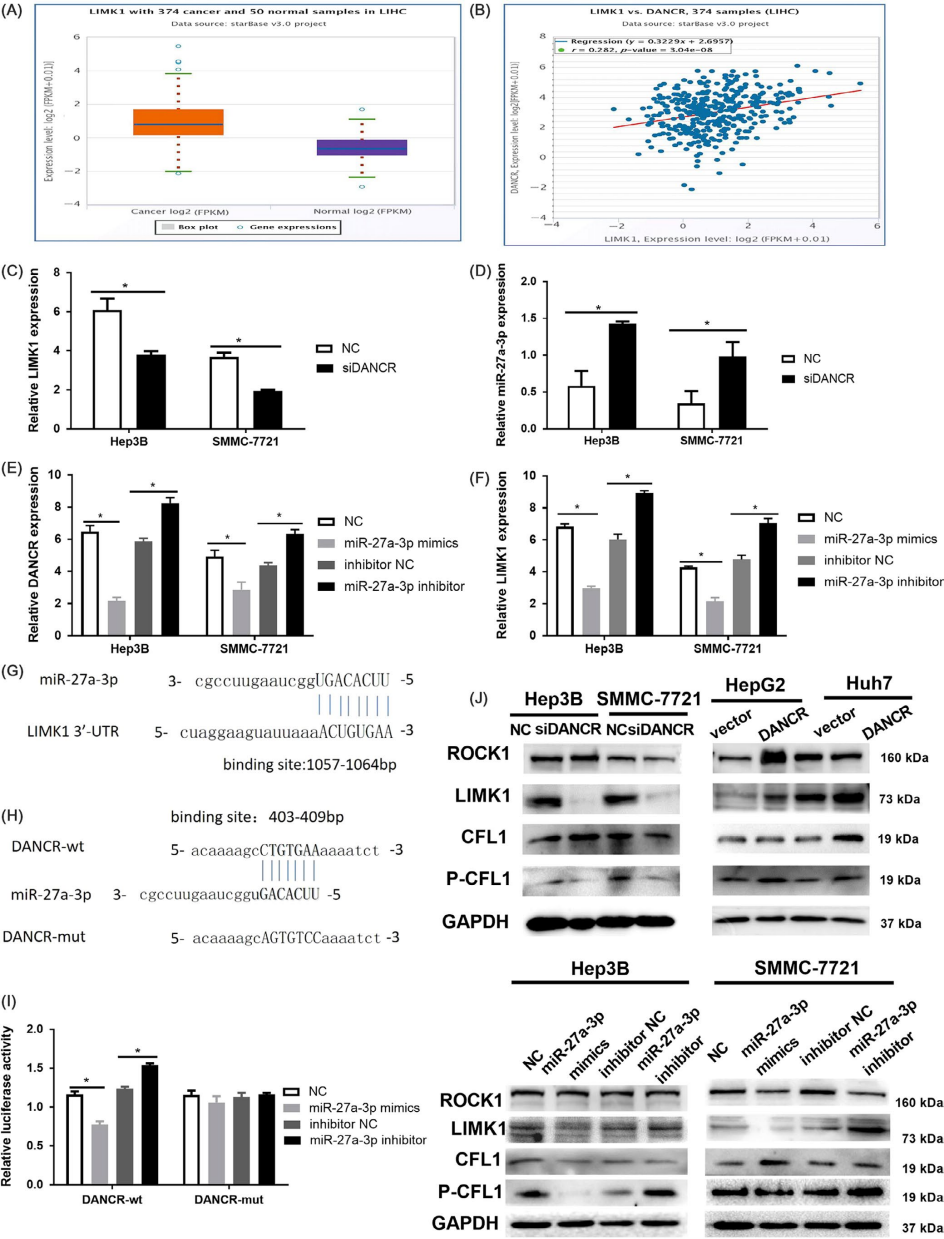
DANCR acts as a sponge of miR‐27a‐3p to regulate LIMK1. A and B, The result of analysis on starbase 3.0 predicted the overexpression of LIMK1 in HCC (*P* < 0.05) and the positive relation of DANCR and LIMK1. r = 0.282, *P* < 0.01. C, The result of qRT‐PCR revealed that the expression of LIMK1 was knockdown in siDANCR group compared with NC group. *P < 0.05. D and E, We detected the interaction between DANCR and miR‐27a‐3p by using qRT‐PCR. The expression of miR‐27a‐3p was increased when DANCR was knocked down. The expression of DANCR was restrained in miR‐27a‐3p mimics group while was elevated in miR‐27a‐3p inhibitor group. *P < 0.05. F, The results of qRT‐PCR indicated that the expression of LIMK1 was repressed by miR‐27a‐3p mimics while accelerated by miR‐27a‐3p inhibitor. *P < 0.05. G, The results from targetscan 7.1 showed the binding site between miR‐27a‐3p and LIMK1 3′‐UTR. H, The result of analysis on AnnLnc showed the binding site of DANCR and miR‐27a‐3p. We muted the binding site (403‐409 bp) on DANCR to construct DANCR‐mut vector for dual‐luciferase reporter assay. I, The result of dual‐luciferase reporter assay indicated DANCR really interacted with miR‐27a‐3p. Co‐transfection of 293T cells with DANCR‐wt and miR‐27a‐3p mimics decreased the luciferase activity while co‐transfection with DANCR‐wt and miR‐27a‐3p inhibitor accelerated the luciferase activity. However, the effect was completely abolished by DANCR‐mut. *P < 0.05. J, DANCR and miR‐27a‐3p modulated the expression of LIMK1 on protein level. The expression of LIMK1 was attenuated when DANCR was knocked down and enhanced in DANCR group. The expression of LIMK1 was decreased in miR‐27a‐3p mimics group while increased in miR‐27a‐3p inhibitor group. All data were collected by repeating three‐time experiments which were mutually independent as mean ± SD

For profound mechanism research, analysis of starbase 3.0 revealed that LIMK1, LIM domain kinase 1, was overexpressed in HCC was positively correlated with the expression of DANCR in HCC (Figure 5A,B), which were in accordance with the results of gepia analysis using the samples from TCGA database (Figure S4). In addition, the results were further verified using qRT‐PCR. Consistently, downregulation of DANCR reduced the expression of LIMK1 in mRNA level (Figure 5C). More recent studies have revealed that lncRNAs may act as ceRNA by competitively binding with miRNAs in tumorigenesis, and we wonder whether the correlation between DANCR and LIMK1 is modulated by miRNAs. Then, the analyses of annolnc and targetscan 7.1 help us discover the potential miRNAs (Figure S5) and finally we decided the miR‐27a‐3p, which could both interact with DANCR and LIMK1 3′‐UTR as candidate for research following. Next, we validated this assumption in research. qRT‐PCR analysis showed that DANCR knockdown increased the expression of miR‐27a‐3p, and miR‐27a‐3p mimics restrained the expression of DANCR while miR‐27a‐3p inhibitor increased the expression of DANCR (Figure 5D,E). As for LIMK1, the expression was reduced when transfected with miR‐27a‐3p mimics while elevated in miR‐27a‐3p inhibitor group (Figure 5F). In detail, the binding site between miR‐27a‐3p and LIMK1 3′‐UTR, as well as DANCR and miR‐27a‐3p were collected from targetscan 7.1 and annolnc (Figure 5G,H). Then, dual‐luciferase reporter assay was conducted to further confirm the hypothesis. Just as the result shown (Figure 5I), co‐transfection of H293T cells with both DANCR‐wt and miR‐27a‐3p mimics decreased the luciferase activity and co‐transfection with DANCR‐wt and miR‐27a‐3p inhibitor increased the activity. However, transfection of DANCR‐mut abrogated the above effect. The Renilla luciferase activity was regarded as control.

We subsequently investigated the effect of DANCR and miR‐27a‐3p on LIMK1 on protein level using Western blot. Corresponding with the results above, the expression of LIMK1 protein was strikingly decreased when DANCR was knocked down, and in DANCR group, the expression of LIMK1 protein was increased. After being transfected with miR‐27a‐3p mimics, the expression of LIMK1 protein was eliminated while the expression of LIMK1 was elevated in miR‐27a‐3p inhibitor group. In addition, the expression of p‐cofilin 1 (p‐CFL1), which is the only known downstream of LIMK1, responded to LIMK1 while the expression of Rho‐associated kinase 1 (ROCK1) and cofilin 1 (CFL1) was not significantly different (Figure 5J). Taken together, DANCR decoys miR‐27a‐3p to regulate the expression of LIMK1.

### 3.6 miR‐27a‐3p inhibits growth and metastasis of HCC cells

**Figure 6:**
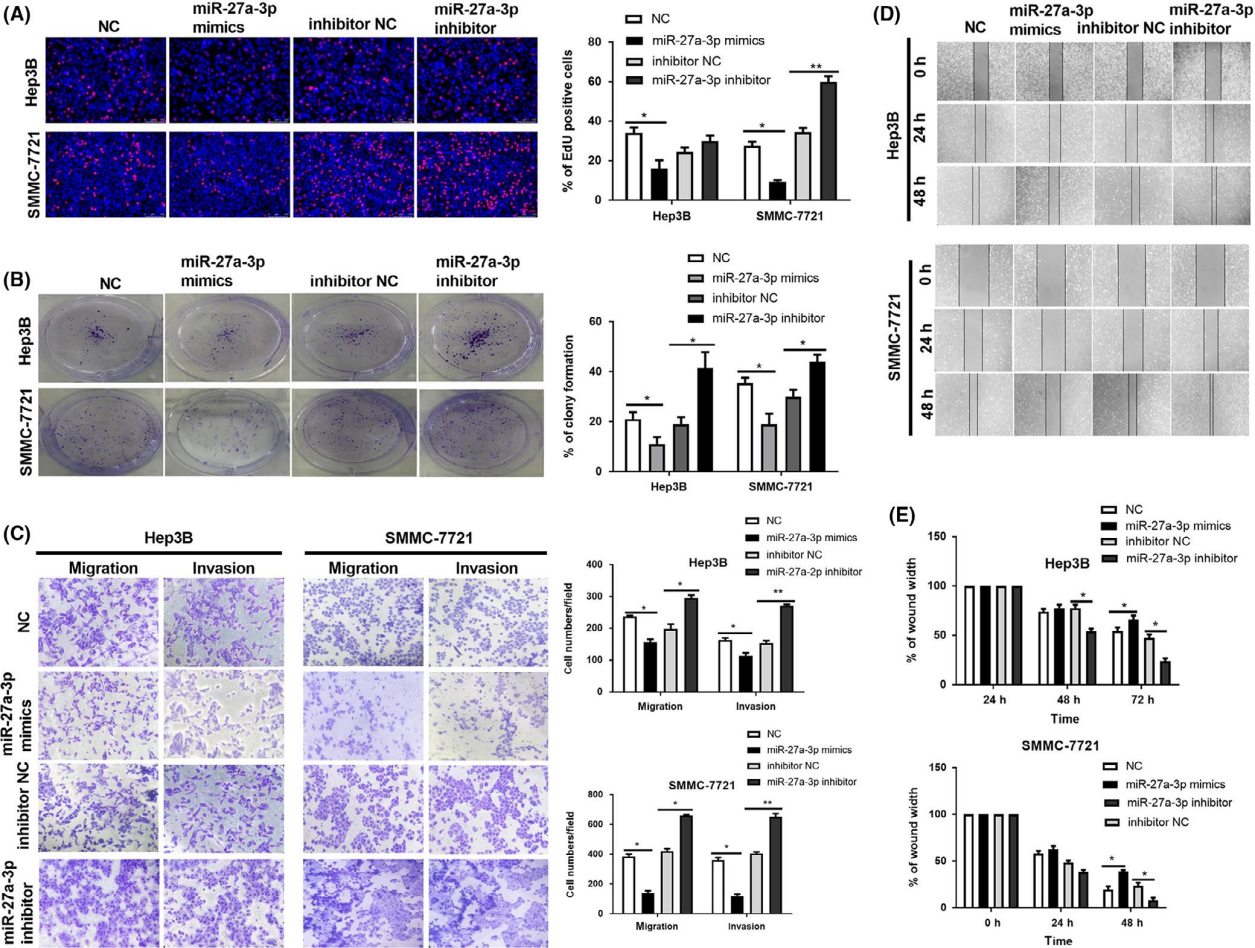
miR‐27a‐3p inhibits growth and metastasis of HCC cells. A, The results of EdU assay revealed that the percentage of EdU‐positive cells decreased in miR‐27a‐3p mimics group and increased in miR‐27a‐3p inhibitor group. *P < 0.05, **P < 0.01. B, In clone formation assay, miR‐27a‐3p mimics inhibited clone formation of HCC cells while miR‐27a‐3p inhibitor promoted clone formation. *P < 0.05. C, In transwell assay, the migration and invasion capacity of HCC cells was reduced by miR‐27a‐3p mimics and apparently enhanced by miR‐27a‐3 p inhibitor. *P < 0.05, **P < 0.01. D and E, The results of wound healing assay showed that HCC cells moved slower in miR‐27a‐3p mimics group while miR‐27a‐3p inhibitor obviously expedited the mobility of HCC cells. *P < 0.05. All data were collected by repeating three‐time experiments which were mutually independent as mean ± SD

In our research, we detected the function of miR‐27a‐3p in vitro to verify its repression on HCC progression. Firstly, the results of EdU assay revealed that the number of EdU‐positive cells decreased in miR‐27a‐3p mimics group while increased in miR‐27a‐3p inhibitor group (Figure 6A). Similarly, clone formation assay indicated that miR‐27a‐3p mimics repressed the clone formation of HCC cells while miR‐27a‐3p inhibitor improved clone formation capacity of HCC cells (Figure 6B). miR‐27a‐3p mimics evidently inhibited HCC cells migration and invasion in transwell assay while miR‐27a‐3p inhibitor severely advocated the migration and invasion of HCC cells (Figure 6C). Considering wound healing assay, miR‐27a‐3p mimics obviously attenuated HCC cells mobility and cells in miR‐27a‐3p inhibitor group migrated more quickly than that in inhibitor NC group (Figure 6D,E). In short, miR‐27a‐3p restrains HCC cell proliferation and metastasis in vitro.

### 3.7 miR‐27a‐3p reverses the effect of DANCR on HCC development by regulating LIMK1

**Figure 7:**
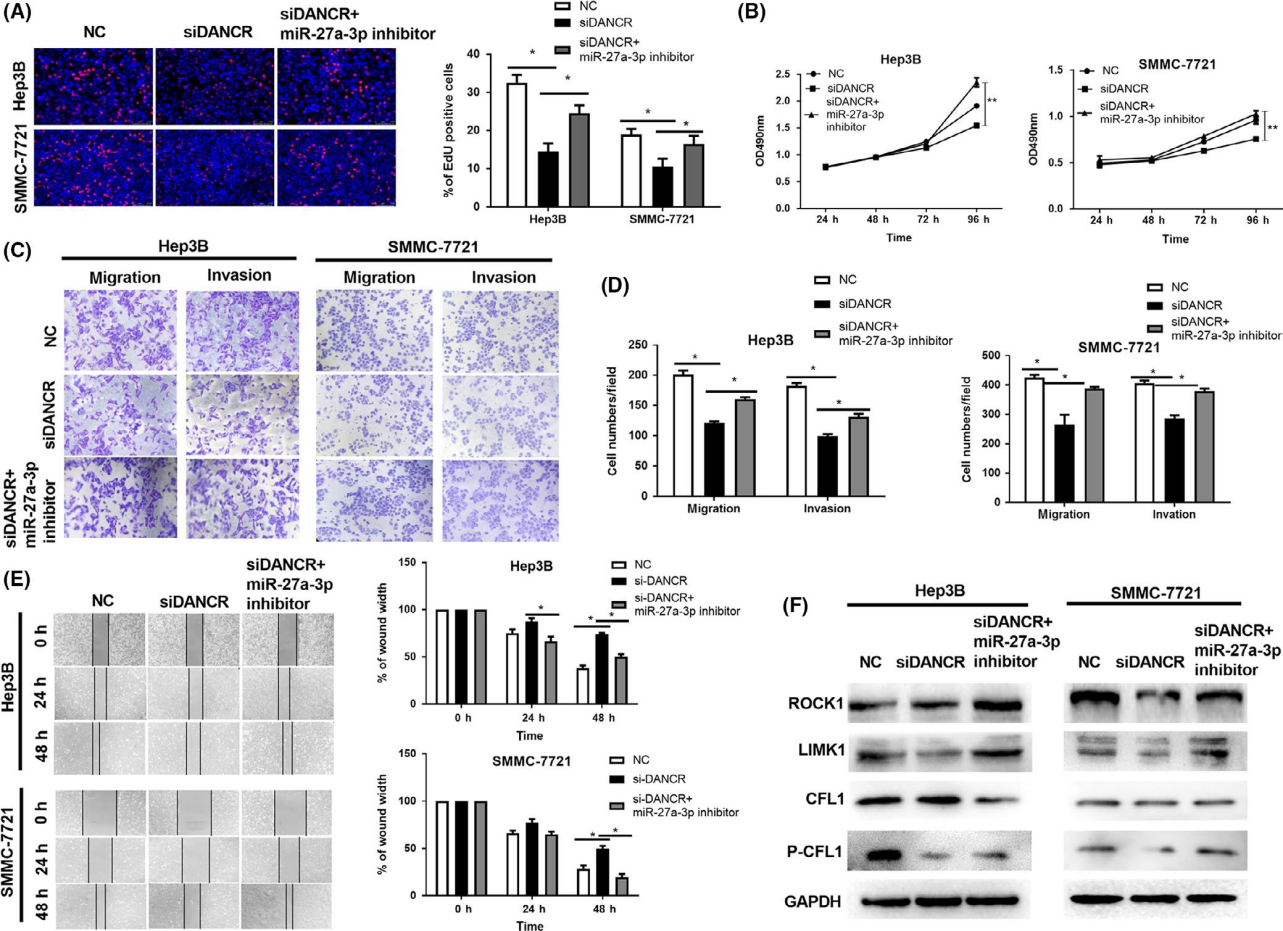
miR‐27a‐3p reverses the effect of DANCR on HCC development by regulating LIMK1. A, The results of EdU assay showed that the percentage of EdU‐positive cells increased by miR‐27a‐3p inhibitor compared with siDANCR group. *P < 0.05. B, In MTT assay, miR‐27a‐3p inhibitor restored the inhibition of siDANCR on cell proliferation over time. HCC cells grew faster in siDANCR and miR‐27a‐3p inhibitor co‐transfection group than siDANCR group. **P < 0.01. C and D, The migration and invasion capacity restrained by downregulation of DANCR could be reversed by miR‐27a‐3p inhibitor. Cells migrated and invaded were increased in siDANCR and miR‐27a‐3p inhibitor co‐transfection group. *P < 0.05. E, In wound healing assay, miR‐27a‐3p inhibitor accelerated the moving of HCC cells compared with siDANCR group. F, In protein level, the downregulation of LIMK1 and P‐CFL1 regulated by siDANCR could be rescued by miR‐27a‐3p inhibitor. All data were collected by repeating three‐time experiments which were mutually independent as mean ± SD

Results mentioned above indicated that DANCR promotes HCC progression by decoying miR‐27a‐3p to regulate LIMK1. We thus detected the effect of DANCR combined with miR‐27a‐3p. Interestingly, the percentage of EdU‐positive cells was increased in co‐transfection with siDANCR and miR‐27a‐3p inhibitor group compared with siDANCR group (Figure 7A). Identically, miR‐27a‐3p inhibitor abrogated the repression of siDANCR on cell growth over time in MTT assay (Figure 7B). Transwell assay showed that miR‐27a‐3p inhibitor apparently increased the migration and invasion capacity of HCC cells when compared with siDANCR group (Figure 7C,D). In wound healing assay, HCC cells moved strikingly faster in siDANCR and miR‐27a‐3p inhibitor group when compared with siDANCR only group (Figure 7E). In protein level, the inhibition of siDANCR on LIMK1 and P‐CFL1 could be restored by miR‐27a‐3p inhibitor (Figure 7F). In conclusion, miR‐27a‐3p inhibitor rescues the inhibition of DANCR knockdown on HCC progression.

### 3.8 DANCR/miR‐27a‐3p axis regulates EMT progression

**Figure 8:**
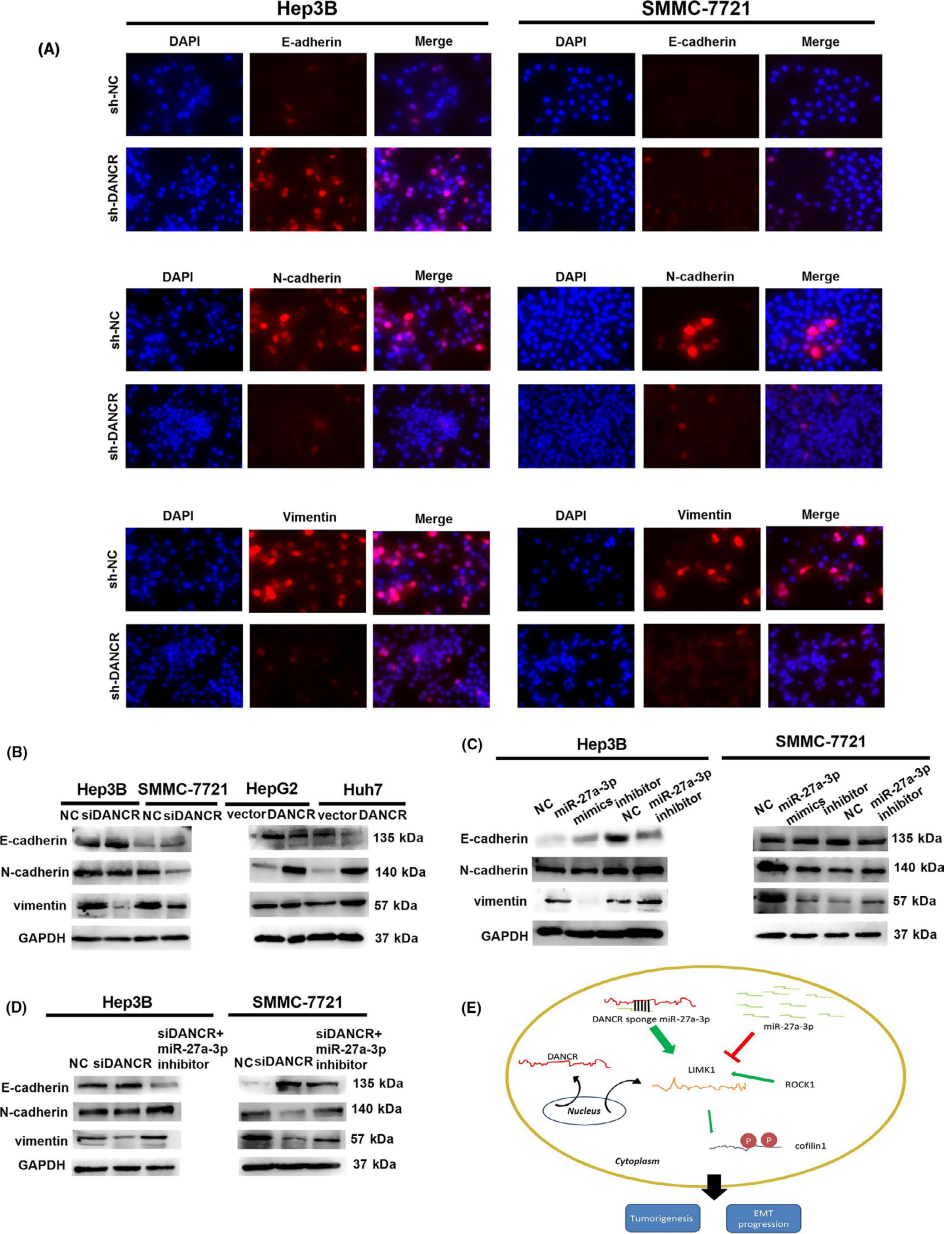
DANCR/miR‐27a‐3p axis regulate EMT progression. A, EMT relevant biomarkers were detected using cell immunofluorescence and the results indicated that the expression of the epithelial marker E‐cadherin was enhanced and the mesenchymal markers N‐cadherin and vimentin were attenuated when DANCR was knocked down. B, The results of Western blot showed that the epithelial marker E‐cadherin was increased while mesenchymal markers N‐cadherin and vimentin were decreased in siDANCR group compared with NC group. When DANCR was overexpressed, Epithelial marker E‐cadherin was decreased while mesenchymal markers N‐cadherin and vimentin was increased. C, MiR‐27a‐3p mimics augmented the expression of E‐cadherin and reduced the expression of N‐cadherin and vimentin. MiR‐27a‐3p inhibitor had the opposite effect on EMT. D, miR‐27a‐3p inhibitor restrained the promotion of siDANCR on E‐cadherin and rescued the inhibition of siDANCR on N‐cadherin and vimentin. E, Schematic diagram of DANCR on hepatocellular carcinoma development in this research. All data were collected by repeating three‐time experiments which were mutually independent as mean ± SD

Epithelial‐mesenchymal transition (EMT) has been reported to play an important role in carcinoma metastasis. We confirmed that DANCR/miR‐27a‐3p axis mediated HCC cells migration and invasion, so we speculated that DANCR/miR‐27a‐3p axis induces epithelial‐mesenchymal transition (EMT) progression. Firstly, cell immunofluorescence was applied to investigate the EMT relevant markers. As the result shown, the expression of the epithelial marker E‐cadherin was enhanced and the mesenchymal markers N‐cadherin and vimentin were attenuated when DANCR was knocked down (Figure 8A). Meanwhile, we also detected EMT relevant markers in protein level. Consistent with the results of cell immunofluorescence, the epithelial marker E‐cadherin was increased while the mesenchymal markers N‐cadherin and vimentin were decreased in siDANCR group when compared with NC group. On the other hand, overexpression of DANCR had the opposite effect on EMT relevant markers (Figure 8B).

As for the effect of miR‐27a‐3p on EMT, epithelial marker E‐cadherin was increased and mesenchymal biomarkers N‐cadherin and vimentin were decreased in miR‐27a‐3p mimics group and the function of miR‐27a‐3p inhibitor was just inverse (Figure 8C).

In view of DANCR as a sponge of miR‐27a‐3p, we confirmed that miR‐27a‐3p inhibitor reversed the repression on EMT progression caused by downregulation of DANCR. In siDANCR and miR‐27a‐3p inhibitor co‐transfection group, epithelial marker E‐cadherin was decreased when compared with siDANCR group. To some extent, suppression of siDANCR on mesenchymal biomarkers N‐cadherin and vimentin was restored by miR‐27a‐3p inhibitor (Figure 8D).

In a word, DANCR/miR‐27a‐3p axis modulates EMT progression which contributes to HCC metastasis.

## 4 DISCUSSION

Hepatocellular carcinoma (HCC) is the most common primary solid tumour, representing the sixth leading cause of cancer and the third leading cause of cancer‐related mortality, which brings heavy burden for patients.^18^ It is imperative to find reliable biomarkers at the preliminary stage. We found DANCR as an oncogene that promoted HCC development by decoying miR‐27a‐3p via ROCK1/LIMK1/COFILIN1 pathway and mediated EMT progression, which may provide novel prognostic factors and therapeutic targets for HCC.

Long non‐coding RNAs (lncRNAs), long than 200 nucleotides, represent a vital proportion in non‐coding RNAs (ncRNAs).^19^ The flexibility of lncRNAs enables them to interact with proteins, RNA or more unknown molecules directly or indirectly.^19^ Considering their location and distribution in the genome, lncRNAs comprehensively participate in gene regulation, cancer phenotypes, cell differentiation and even chromatin remodelling.^19^, ^20^ It is reported that DANCR, mapped into human chromosome 4q12.5 locus, is more localized to cytoplasm, which powerfully indicates DANCR may influence the transcription of a multitude of proteins via base pairing or forming networks.^10^, ^19^, ^21^ There are also studies confirm that DANCR targeted by MYC aggravates cancer partly by repressing p21^22^; DANCR decoys miR‐335‐5p and miR‐1972 to mediate osteosarcoma development via regulating ROCK1.^23^ In our study, we detected the positive relation of DANCR and LIMK1 and completely demonstrated the function of DANCR in HCC. DANCR aggravates HCC cells proliferation and metastasis meanwhile accelerated EMT procedure. Knockdown of DANCR had the opposite effect.

CeRNA is one of the mechanisms linking seral ncRNAs including lncRNAs, miRNAs and pseudogenes with genes encoding proteins in cancers.^19^ The target molecules regulate mutual expression by competing for the binding of miRNA's response elements (MREs).^24^ For instance, SNHG5 regulates GSK3‐β expression by competitively binding miR‐26a‐5p in HCC.^25^ MEG3 inhibits proliferation and promotes apoptosis of bladder urothelial carcinoma cells by decoying miR‐96 and then indirectly regulates TPM1.^26^ In our study, we demonstrated that DANCR acted as a competing endogenous RNA of miR‐27a‐3p to regulate LIMK1 expression in HCC development.

ROCK1/LIMK1/COFILIN1 pathway has been a well‐understood signal pathway which is reported to regulate actin cytoskeletal dynamics, and thus influences the survival and metastasis of cancer cells.^27^, ^28^ LIMK1 is a serine/threonine kinase that regulates actin polymerization via phosphorylation and inactivation of the actin‐binding factor cofilin (CFL), which is the only known substrate of LIMK at the present. As a gene on the upstream of LIMK1, Rho‐associated kinase (ROCK) is responsible for the activation of LIMK1 at Thr‐508.^27^, ^28^, ^29^, ^30^ In the present study, we found that LIMK1 was one of the target genes of miR‐27a‐3p and positively correlates to the expression of DANCR. The results further indicated that DANCR promoted HCC progression as a sponge for miR‐27a‐3p to regulate ROCK1/LIMK1/CFL1 pathway.

## 5 CONCLUSIONS

In conclusion, DANCR promotes HCC development and mediates EMT by decoying miR‐27a‐3p and regulating ROCK1/LIMK1/COFILIN pathway. Our study firstly connected DANCR, miR‐27a‐3p and LIMK1 together and investigated their function in HCC thoroughly. However, further verification by a large number of clinical samples is still required. Our research maybe a little part of the complicated mechanism on tumorigenesis of HCC, but it is worthwhile to reveal that DANCR may be a new molecular target for clinical diagnosis and treatment of HCC.^12^

## CONFLICT OF INTEREST

The authors declare that they have no conflict of interest.

## AUTHOR CONTRIBUTIONS

DG and YL conceived and designed the study. YC and DZ help perform the experiments and analysed the data. GL, MR and XL assisted the experiments. YL, XW and SH revised the manuscript which was written by DG. All authors have read and approved the final manuscript.
