# Supplementary material for: DANCR promotes HCC progression and regulates EMT by sponging miR‐27a‐3p via ROCK1/LIMK1/COFILIN1 pathway
Source: Cell Prolif. 2019 Apr 30;52(4):e12628. doi: 10.1111/cpr.12628 (PMC6668976; doi:10.1111/cpr.12628)
Supplement: Supplementary file 1 [file CPR-52-e12628-s001.docx]

**Supplementary figures**


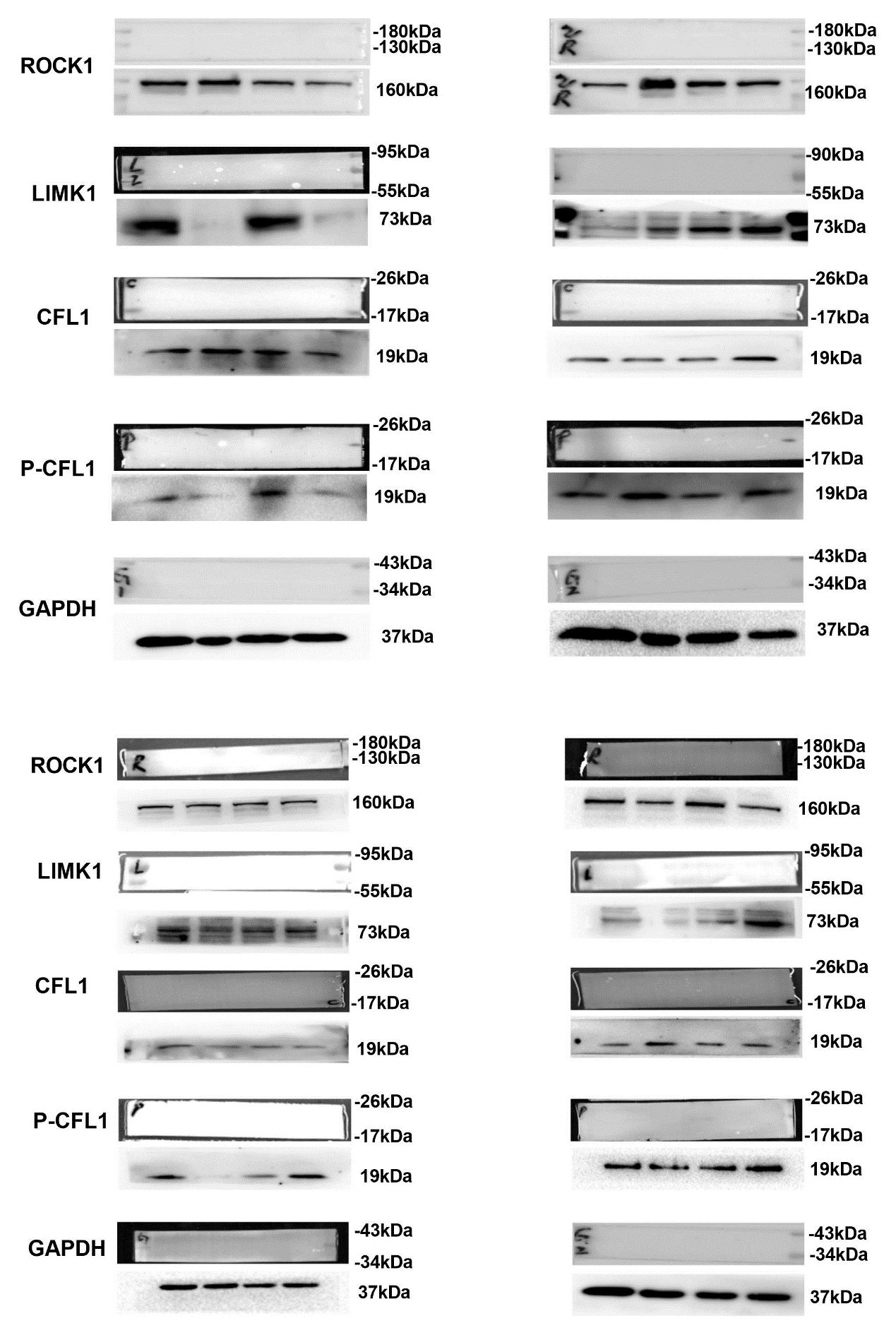


**Supplementary figure 1.** The raw data for western blot in Fig. 5J.


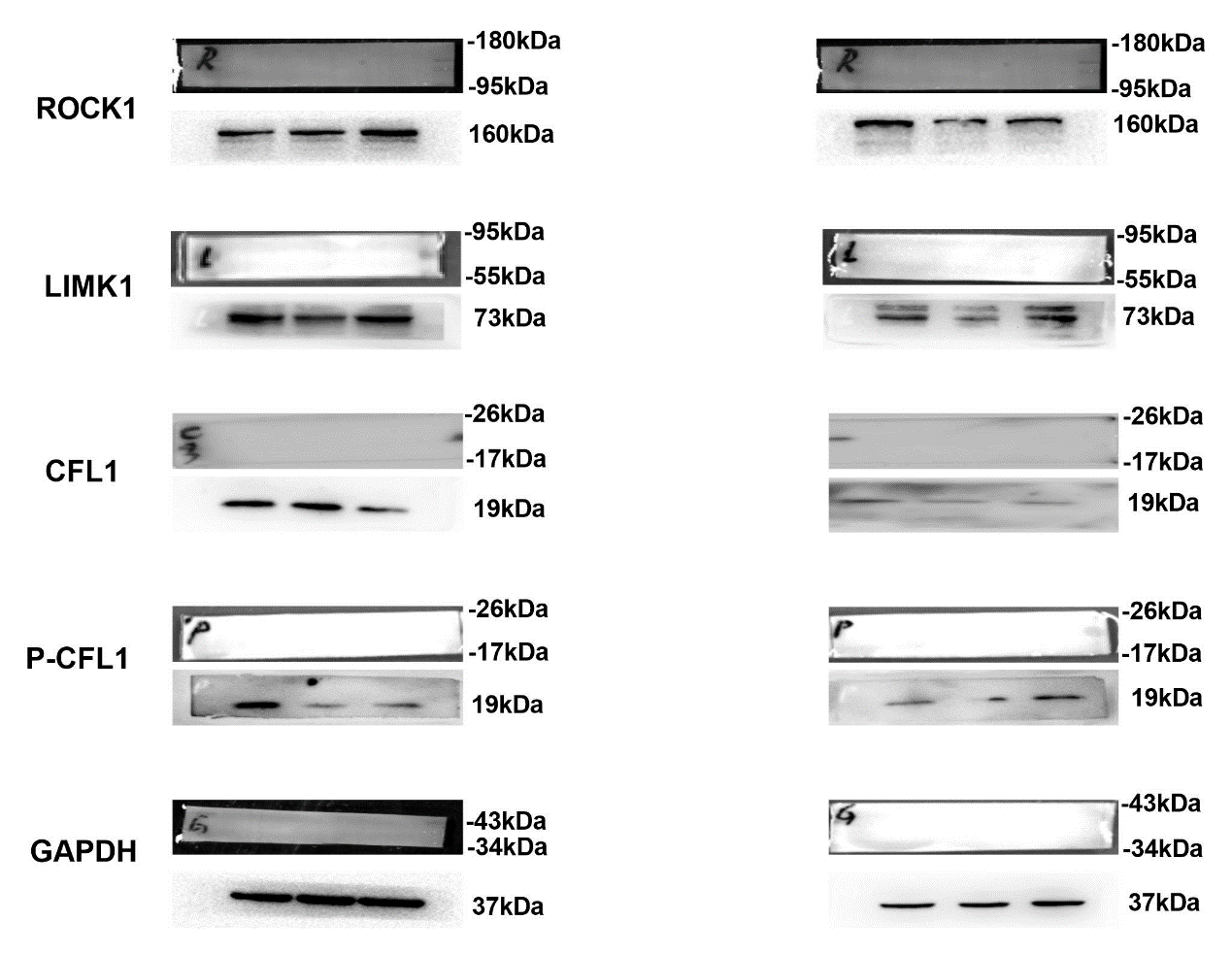


**Supplementary figure 2.** The raw data for western blot in Fig. 7F.


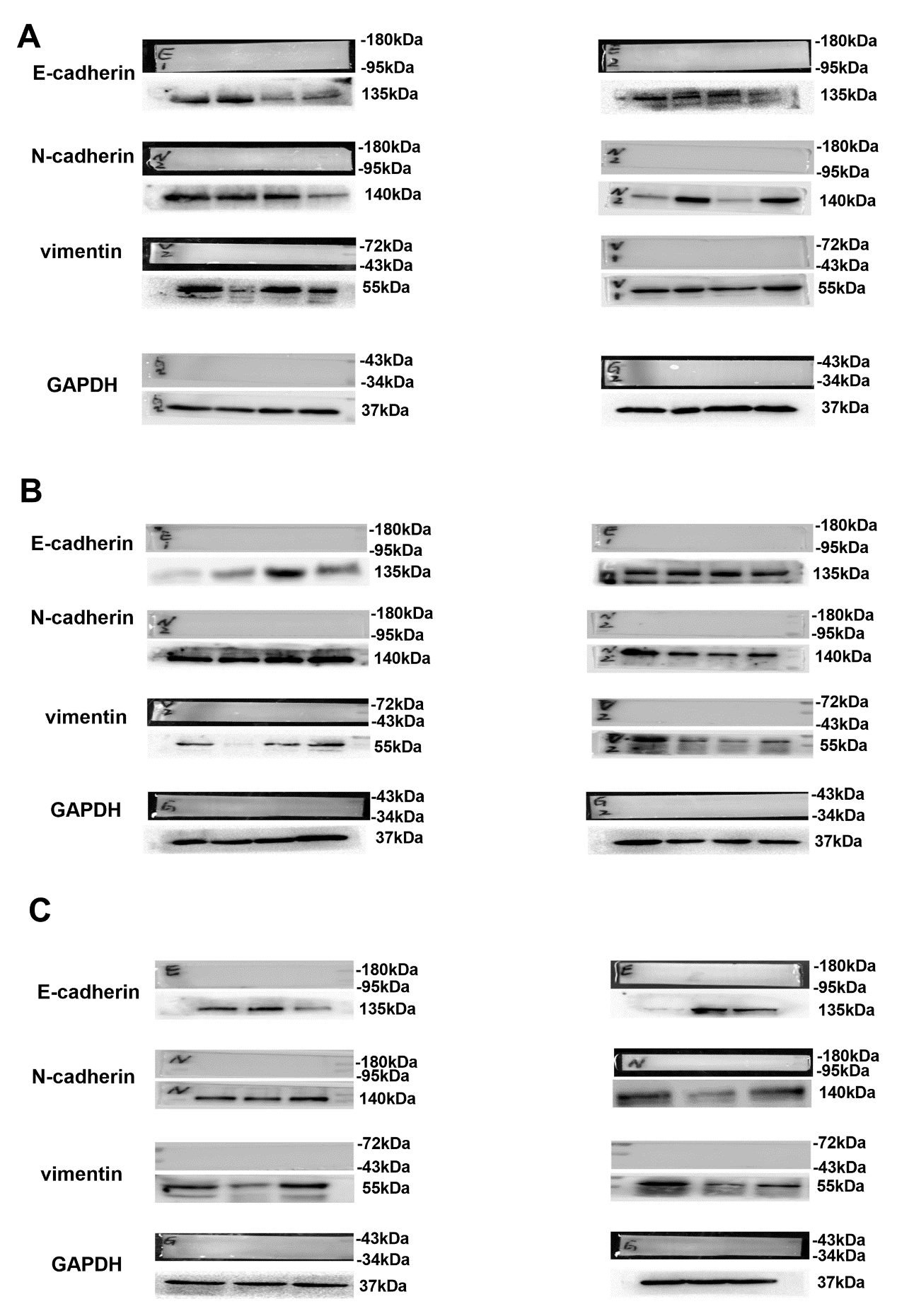


**Supplementary figure 3.** **a-c.** The raw data for western blot in Fig. 8B, C, and D one by one.


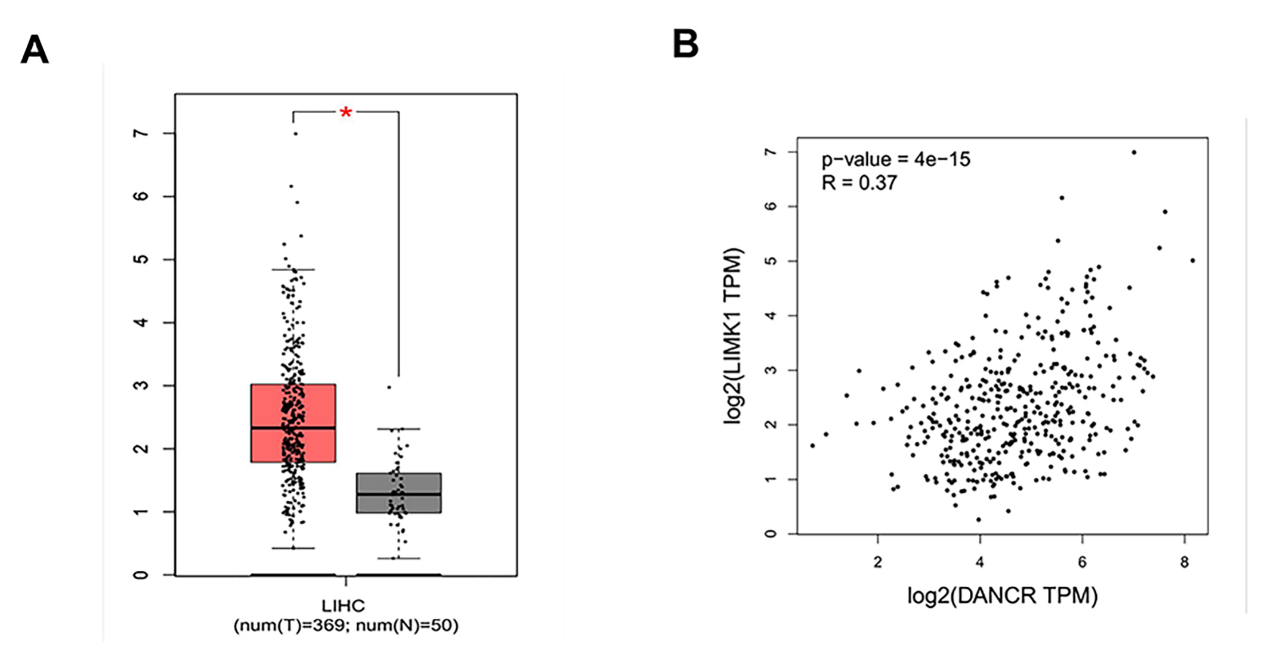


**Supplementary figure 4.** The results of GEPIA by analyzing samples from TCGA database showed that the high expression of LIMK1 in HCC (**p*<0.05) and the positive correlation of DANCR and LIMK1 (*p*<0.01, R=0.37).


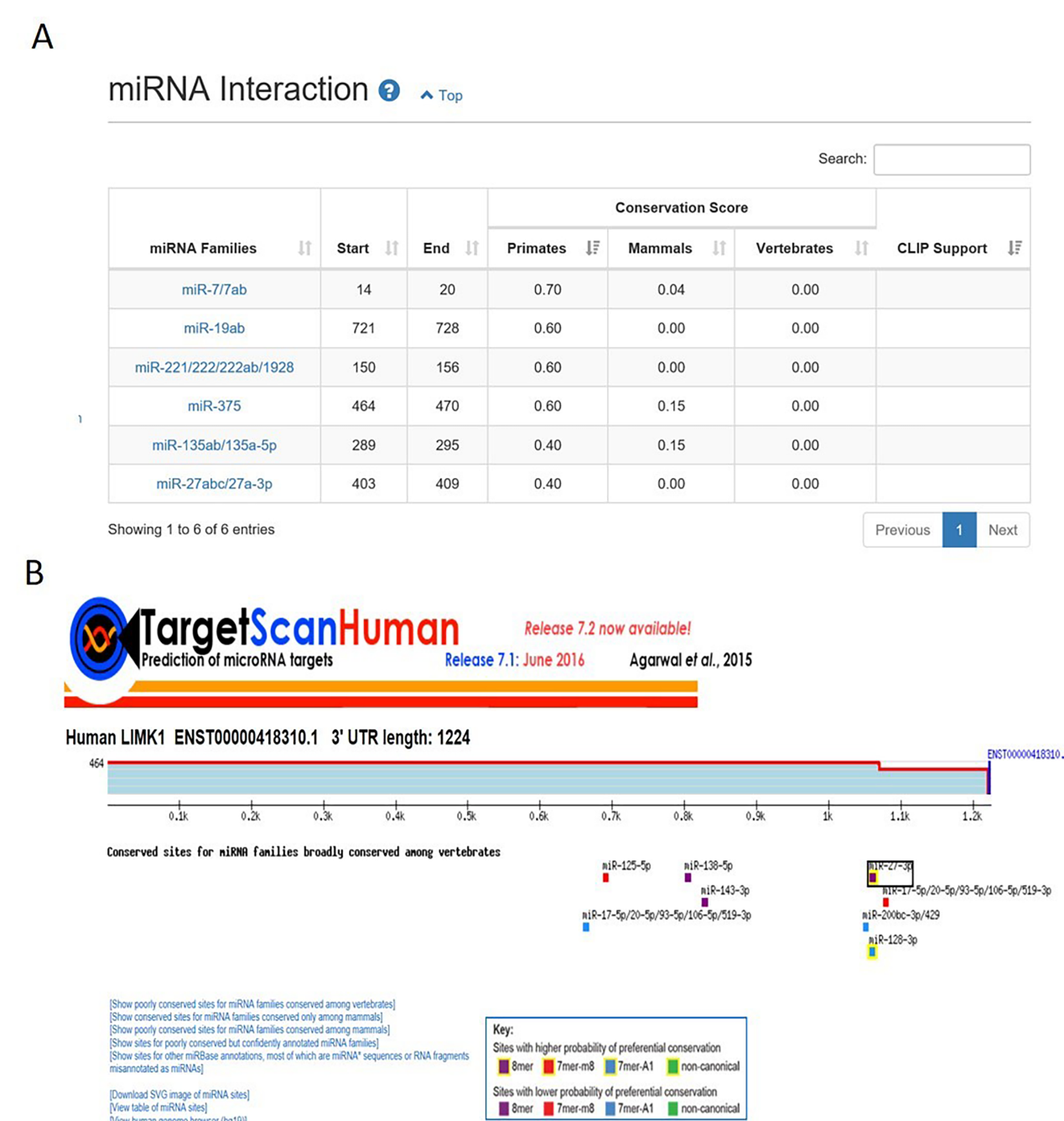


**Supplementary figure 5. a.** Analysis of AnnoLnc indicates the miRNAs that interact with DANCR. **b.** the results of analysis on TargetScan 7.1 shows the miRNAs that could interact with LIMK1 3’-UTR.
